# Homeometric autoregulation in severe aortic stenosis: insights from transcatheter aortic valve replacement

**DOI:** 10.3389/fcvm.2025.1677372

**Published:** 2026-01-12

**Authors:** Adam J. Doerr, Matthew Gottbrecht, Nikolaos Kakouros, Matthew W. Parker, Colleen M. Harrington, Gerard P. Aurigemma

**Affiliations:** 1 Division of Cardiovascular Medicine, Department of Medicine, UMass Chan Medical School, Worcester, MA, United States; 2 Division of Cardiovascular Medicine, Department of Medicine, Saint Vincent Hospital, Worcester, MA, United States; 3 Division of Cardiology, Department of Medicine, Harvard Medical School, Boston, MA, United States

**Keywords:** aortic stenosis, homeometric autoregulation, ventricular adaptation, transcatheter aortic valve replacement, echocardiography

## Abstract

**Background:**

In severe aortic stenosis (AS), relief of afterload excess would be expected to improve left ventricular ejection fraction. However, the response of LVEF to transcatheter aortic valve replacement (TAVR) is variable, with some patients even demonstrating a decline. The mechanisms underlying this phenomenon are incompletely characterized. Accordingly, we investigated changes in systolic function in the near-term postoperative period following TAVR.

**Methods:**

We studied consecutive patients with severe AS referred for TAVR without identifiable perioperative sources of negative inotropy or ventricular dyssynchrony. Preoperative and postoperative day one echocardiograms were compared with respect to hemodynamics, LV geometry, LVEF, and midwall fractional shortening (FSmw). Contractility was assessed by comparing observed FSmw values to those predicted based on the stress-shortening relation of healthy controls.

**Results:**

Thirty-six patients were included (61% women; mean age 77 years; mean Society of Thoracic Surgeons mortality risk score 3.6%). Following TAVR, there was a precipitous decline in circumferential end-systolic wall stress from 122 ± 47 to 74 ± 32 kdyn/cm^2^ (*p* < 0.001) and a slight increase in LVEF. Surprisingly, however, there was also an increase in the percentage of patients with depressed contractility from 22% (8) to 78% (28) (*p* < 0.001). Heart rate and ventricular volumes remained unchanged.

**Conclusions:**

Contractility declined in the near-term postoperative period following TAVR. We interpret this finding to suggest that contractility is augmented by high afterload in severe AS and declines in parallel with afterload reduction. We speculate that autoregulatory mechanisms triggered by high valvular resistance support LVEF in severe AS and rapidly abate following TAVR.

## Introduction

In severe aortic stenosis (AS), left ventricular systolic function is maintained through the development of concentric hypertrophy or remodeling (1, 2), wherein the parallel addition of sarcomeres increases wall thickness relative to chamber size (3). This process mitigates the adverse effect of excess end-systolic wall stress caused by high valvular resistance (4). It is generally assumed that reduced LVEF in severe AS without coronary artery disease results from afterload excess (4). However, data from studies employing midwall stress-shortening relations suggest some patients with severe AS also have abnormalities in LV contractility (5). Such reduced contractility appears to result from replacement fibrosis and microvascular dysfunction (6, 7).

Transcatheter aortic valve replacement (TAVR) represents a unique opportunity to study LV mechanics in severe AS due to the immediate drop in afterload and because the confounding effects of thoracotomy and cardioplegia-related myocardial depression are obviated. While pooled results from several large trials suggest an inverse relationship between baseline LVEF and the magnitude of LVEF improvement following TAVR (8–21), recent work has demonstrated a lack of significant improvement or even a decline in LVEF following TAVR in patients with baseline LVEF >60% (22). This occurred despite a decrease in afterload as assessed by both wall stress and valvuloarterial impedance. Barring significant changes in preload, which is thought to only have a modest impact on LVEF at the extremes (23), these findings suggest contractility decline. However, given the 1-year interval between TAVR and repeat echocardiography in that study, it is difficult to discount a reduction in contractility through earlier and faster regression of myocyte hypertrophy than interstitial fibrosis (24, 25). While subsequent work demonstrated similar findings in the immediate postoperative period (26), the use of LVEF, even when indexed to wall stress, fails to account for LV concentric remodeling, which inherently confounds the assessment of contractility (1, 27).

This study aimed to characterize the near-term, isolated effect of TAVR on LV contractility in the absence of confounding effects from loading conditions, geometric abnormalities, and common perioperative sources of negative inotropy encountered in this population.

## Methods

### Patient selection

This was a single-center retrospective cohort study. Patients were considered for inclusion if they had severe AS as defined by contemporary guidelines (28), underwent TAVR at UMass Memorial Medical Center between 1 January 2021 and 29 September 2021, and had preoperative echocardiograms available for review if performed at another institution. The following exclusion criteria were sequentially applied: previous transcatheter or surgical aortic valve replacement; concomitant non-AS severe valvular disease; myocardial infarction (MI), coronary artery bypass grafting, or percutaneous coronary intervention (PCI) within 8 weeks of TAVR or between pre- and postoperative echocardiograms; unrevascularized hemodynamically significant coronary artery disease (defined as vessel diameter reduction ≥80% or fractional flow reserve <0.8 on coronary angiography); initiation or dose escalation of negative inotropic agents between pre- and postoperative echocardiograms; new conduction abnormality or pacemaker dependence between pre- and postoperative echocardiograms; incomplete clinical data; and inadequate echocardiogram image quality. The UMass Chan Institutional Review Board approved the study and waived the requirement for informed consent due to the retrospective design and minimal potential harm to subjects.

### Outcomes

The primary endpoint was a change in LV contractility based on the stress-shortening relation, as described below. The secondary endpoints included changes in LVEF, circumferential end-systolic wall stress (cESS), valvuloarterial impedance (Zva), and mean AV gradient (AVG).

### Echocardiography

All patients underwent comprehensive two-dimensional and Doppler echocardiography preoperatively and on postoperative day 1 according to American Society of Echocardiography standards (28). Valvuloarterial impedance was calculated as the sum of systolic blood pressure (SBP) and mean AVG divided by stroke volume indexed to body surface area. Left ventricular end-diastolic and end-systolic volumes (EDV and ESV) and LVEF were measured by the biplane disk summation method.

Two-dimensional measurements of septal thickness, LV internal diameter, and LV posterior wall thickness were obtained at the level of the mitral valve leaflet tips in the parasternal long-axis view at end-diastole and end-systole. Midwall fractional shortening and cESS were derived based on a two-shell model of the LV, assuming constant volumes of each shell throughout the cardiac cycle, as previously described (29, 30). Left ventricular mass was calculated using the Devereux equation (cube method) (31, 32).

### Clinical data

Average blood pressure values were derived using readings taken ±2 h from the time of echocardiography to decrease the influence of outliers in afterload calculations. When available, arterial catheter data were used instead of sphygmomanometer measurements. Reported heart rate values were recorded during patients' parasternal long-axis views on echocardiograms. Demographic, anthropomorphic, and comorbidities data were abstracted from the electronic medical record.

### Control cohort

A control cohort from a prior study (33) of midwall dynamics, comprised of 30 patients (43% women, mean age 74 ± 6 years) with normal echocardiograms, was used to derive a control stress-shortening relation through linear regression, defined as FSmw = 28.486 − 0.104 × cESS. The 95% confidence interval lower bound equation was FSmw = 22.702 − 0.106 × cESS.

### Contractility calculations

Contractility was assessed by comparing observed to predicted FSmw based on the control stress-shortening relation. Abnormal contractility was defined as observed FSmw greater than two standard deviations below predicted.

### Statistical analysis

Categorical variables are reported as percentages with absolute values and, when applicable, compared before and after TAVR using McNemar's test. Continuous variables are reported as means with standard deviations and compared before and after TAVR using the paired sample *t*-test if normally distributed or the Wilcoxon signed-rank test if not normally distributed. Normality was assessed by the Shapiro–Wilk test. *P*-values < 0.05 were considered statistically significant. The corresponding author had full access to all the data in the study and takes responsibility for its integrity and the data analysis.

## Results

### Patient characteristics

Among the 100 patients eligible for inclusion, 4 were excluded due to prior surgical or transcatheter aortic valve replacement, 1 due to interval MI, 9 due to interval PCI, 4 due to unrevascularized coronary artery disease, 15 due to new conduction abnormality or pacemaker dependence, 5 due to initiation or uptitration of negative inotropic agents, 1 due to an intraprocedural complication (iatrogenic aorta–RV fistula), 9 due to incomplete clinical data, and 16 due to suboptimal image quality. Therefore, our cohort consists of 36 patients (61% women) with a mean age of 77 ± 9 years and a mean Society of Thoracic Surgeons (STS) mortality risk score of 3.6 ± 2.3%. Additional clinical characteristics are summarized in Table 1. The preoperative rhythm was sinus in 29 patients (81%), atrial fibrillation (AF) in 6 patients (17%), and unclear in 1 patient (3%). There were no missing data for any included patients.

**Table 1:** Baseline demographics and comorbidities.

Characteristic	*N* = 36
Age (years)	77 ± 9
Women	61% (22)
Height (inches)	65 ± 4
Weight (lbs)	165 ± 47
BSA (m^2^)	1.83 ± 0.29
BMI (kg/m^2^)	27.8 ± 7.8
STS score (%)	3.6 ± 2.3
Severe AS subtype	
HG	75% (27)
Classical LFLG	8% (3)
Paradoxical LFLG	6% (2)
NFLG	11% (4)
NYHA class	
II–IV	89% (32)
II	36% (13)
III	44% (16)
IV	8% (3)
Hypertension	94% (34)
Diabetes mellitus	25% (9)
Prior MI	8% (3)
Prior PCI	6% (2)
Prior CABG	6% (2)
AF paroxysmal	9% (3)
AF persistent	31% (11)
Conduction defect	14% (5)
Pacemaker pre-TAVR	11% (4)
CVA	8% (3)
TIA	8% (3)
Chronic lung disease	19% (7)
ESRD	3% (1)
Valve type	
Balloon-expandable	92% (33)
Self-expanding	8% (3)

BSA, body surface area; BMI, body mass index; STS, Society of Thoracic Surgeons; HG, high gradient; LFLG, low flow, low gradient; NFLG, normal flow, low gradient; NYHA, New York Heart Association; MI, myocardial infarction; PCI, percutaneous coronary intervention; CABG, coronary artery bypass grafting; AF, atrial fibrillation; CVA, cerebrovascular disease; TIA, transient ischemic attack; ESRD, end-stage renal disease.

### Echocardiographic and hemodynamic changes

**Figure 1:**
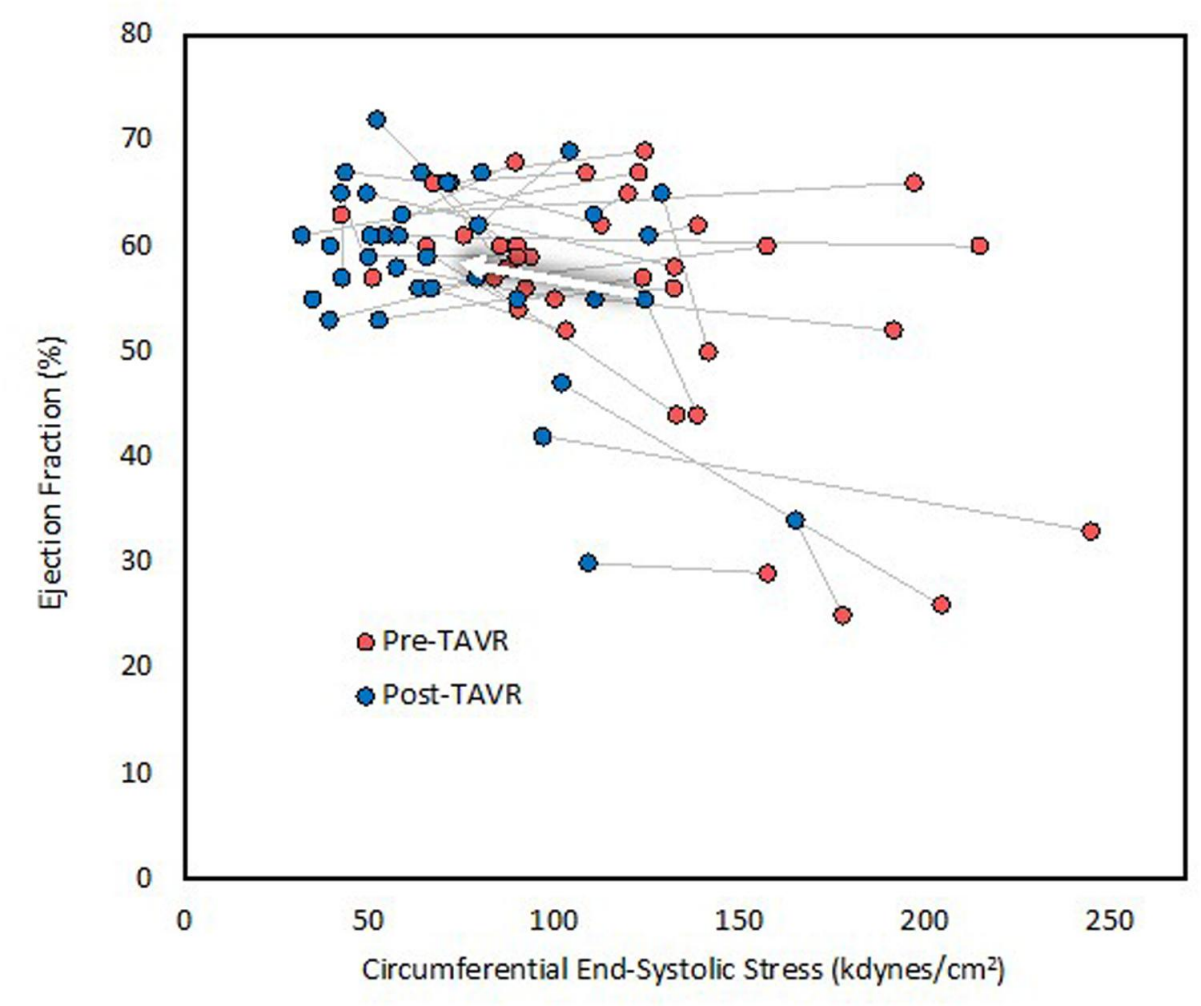
Left ventricular ejection fraction before and after TAVR. LVEF increased only marginally despite significant afterload reduction. The arrow indicates average change.

**Figure 2:**
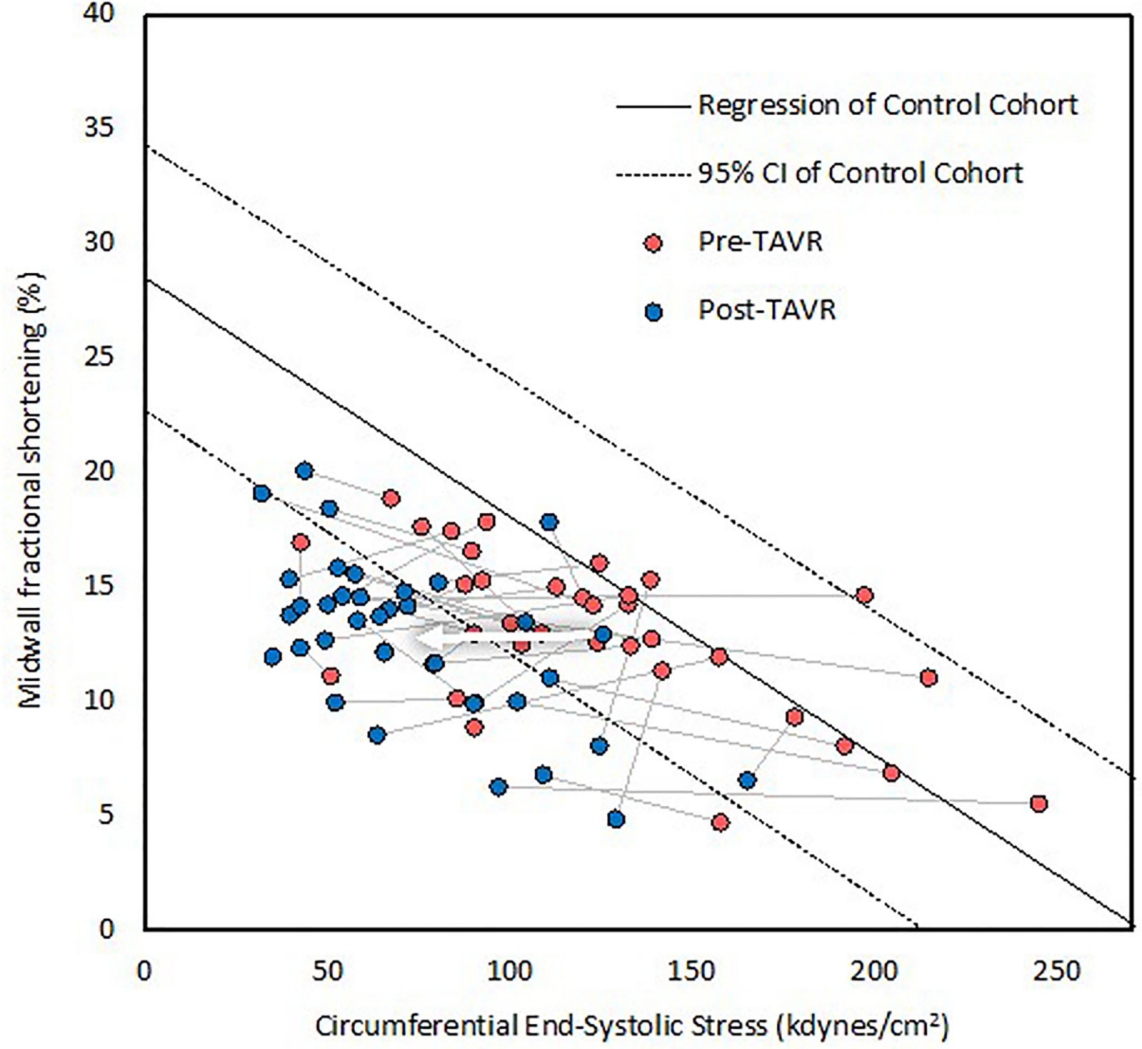
The stress-shortening relationship before and after TAVR. Each patient is represented by a line, with red dots indicating preoperative values and blue dots postoperative values. The dots falling within the 95% CI represent normal contractility. This graph demonstrates that contractility was impaired in many patients at baseline and, on average, declined further following TAVR, as evidenced by horizontal movement crossing the lower bound 95% confidence interval of normal. The arrow indicates average change. CI indicates confidence intervals.

The mean length of time between preoperative echocardiogram and TAVR was 92 ± 89 days (range 3–472 days), and the mean length of time between TAVR and postoperative echocardiogram was 22 ± 4 h (range 5–25 h). Echocardiographic and hemodynamic parameters before and after TAVR are summarized in Table 2. SBP remained unchanged at 135 mmHg, whereas diastolic blood pressure decreased significantly from 72 ± 8 to 58 ± 12 mmHg. Aortic valve area increased significantly, whereas the mean and peak AVG decreased significantly. Circumferential ESS decreased significantly from 122 ± 47 to 74 ± 32 kdyn/cm^2^, as well as valvuloarterial impedance from 5.25 ± 1.84 to 3.89 ± 0.87 mmHg/mL/m^2^. End-diastolic volume, end-systolic volume, and stroke volume index remained unchanged, whereas LVEF increased significantly from 55 ± 11% to 58 ± 9% (Figure 1). The percentage of patients with FSmw greater than two standard deviations below predicted increased significantly from 22% (8) to 78% (28) (Figure 2), whereas the average percent predicted FSmw decreased significantly from 89 ± 33% to 61 ± 15%. Similar decreases in average percent predicted FSmw were observed in patients with high (94 ± 34% to 63 ± 16%) vs. low (73 ± 24% to 55 ± 10%) mean AVG, normal (90 ± 36% to 58 ± 17%) vs. low (87 ± 29% to 66 ± 9%) stroke volume index, and preserved (88 ± 31% to 65 ± 14%) vs. reduced (90 ± 39% to 52 ± 12%) LVEF. There was not a strong association between preoperative cESS and the degree of postoperative decline in percent predicted FSmw (*R*^2^ = 0.58).

**Table 2:** Hemodynamic and echocardiographic variables before and after TAVR.

Variable	Pre-TAVR	Post-TAVR	*P*-value	% change
SBP (mmHg)	135 ± 23	132 ± 19	0.40	
DBP (mmHg)	72 ± 8	58 ± 12	<0.001	−19
HR (bpm)	79 ± 15	78 ± 14	0.76	
LVIDd (mm)	45 ± 7	43 ± 7	<0.01	−4
IVSTd (mm)	12 ± 2	13 ± 2	0.14	
PWTd (mm)	12 ± 2	12 ± 2	0.93	
LVIDs (mm)	32 ± 8	30 ± 8	0.07	
LVMI (g/m^2^)	108 ± 33	105 ± 27	0.35	
EDV (mL)	127 ± 38	122 ± 27	0.27	
ESV (mL)	58 ± 29	52 ± 21	0.14	
RWT	0.55 ± 0.14	0.58 ± 0.12	0.07	
AVA (cm^2^)	0.71 ± 0.22	1.58 ± 0.52	<0.001	124
AVAi (cm^2^/m^2^)	0.39 ± 0.13	0.88 ± 0.29	<0.001	122
Mean AVG (mmHg)	48 ± 14	12 ± 6	<0.001	−74
Peak AVG (mmHg)	78 ± 20	20 ± 9	<0.001	−74
cESS (kdyn/cm^2^)	122 ± 47	74 ± 32	<0.001	−39
Zva (mmHg/mL/m^2^)	5.25 ± 1.84	3.89 ± 0.87	<0.001	−26
SVi (mL/m^2^)	38.10 ± 11.45	38.65 ± 8.99	0.72	
LVEF (%)	55 ± 11	58 ± 9	<0.05	5
FSmw (%)	13 ± 3	13 ± 4	0.76	
FSmw percent predicted (%)	89 ± 33	61 ± 15	<0.001	−31
High mean AVG	94 ± 34	63 ± 16	<0.001	−33
Low mean AVG	73 ± 24	55 ± 10	<0.05	−25
Normal baseline SVi	90 ± 36	58 ± 17	<0.001	−36
Low baseline SVi	87 ± 29	66 ± 9	<0.01	−24
Preserved baseline LVEF	88 ± 31	65 ± 14	<0.001	−26
Reduced baseline LVEF	90 ± 39	52 ± 12	<0.05	−42
FSmw ≥2 SD below predicted (%)	22% (8)	78% (28)	<0.001	250

SBP, systolic blood pressure; DBP, diastolic blood pressure; HR, heart rate; LVIDd, left ventricular internal diameter at end-diastole; IVSTd, interventricular septal thickness at end-diastole; PWTd, left ventricular posterior wall thickness at end-diastole; LVIDs, left ventricular internal diameter at end-systole; EDV, end-diastolic volume; ESV, end-systolic volume; RWT, relative wall thickness; AVA, aortic valve area; AVAi, aortic valve area indexed to body surface area; AVG, aortic valve pressure gradient; cESS, circumferential end-systolic wall stress; Zva, valvuloarterial impedance; Svi, stroke volume indexed to body surface area; LVEF, left ventricular ejection fraction; FSmw, midwall fractional shortening.

## Discussion

In this cohort of 36 medically complex patients with severe AS, LVEF was mildly decreased at baseline and increased only slightly following TAVR despite substantial afterload reduction, pointing to an immediate decline in LV contractility. FSmw corrected for cESS was markedly reduced at baseline and decreased further within 24 h of TAVR, in support of this conclusion. Thus, this contractility decline is evident in the immediate post-TAVR period, long before reverse remodeling can take place. It also occurred in the absence of new ischemic insults, pacemaker-related ventricular dyssynchrony, initiation of negative inotropic agents, and confounding effects of thoracotomy. Heart rate remained unchanged, ruling out the contribution from the Bowditch effect (tachycardia-mediated positive inotropy). Therefore, this contractility decline appears to be related to valvular unloading itself.

Accordingly, we hypothesize that systolic function in severe AS is supported by augmentation of contractility in response to high afterload. A similar phenomenon involving tandem increases in effective arterial elastance and ventricular elastance has been demonstrated in hypertensive heart disease and normal aging (34–36). Multiple mechanisms may mediate the interaction between afterload and contractility, including enhanced sympathetic tone and alterations in myocardial calcium handling. In decompensated severe AS, the classic triad of positional or exertional hypotension (37), endomyocardial ischemia (38, 39), and pulmonary venous congestion (40, 41) is a potent trigger of increased adrenergic tone. Remediation of these processes through aortic valve replacement, in turn, reduces adrenergic tone (42). Additionally, acute afterload elevation (e.g., through aortic cross-clamping) has been shown to induce homeometric autoregulation (i.e., the Anrep effect), wherein increased myocardial stretch leads to an increase in the calcium transient amplitude (43–46). Although *in vitro* studies investigating this phenomenon in chronic pressure overload states are lacking, the Anrep effect would provide an elegant connection between valvular stenosis and augmented contractility.

**Figure 3:**
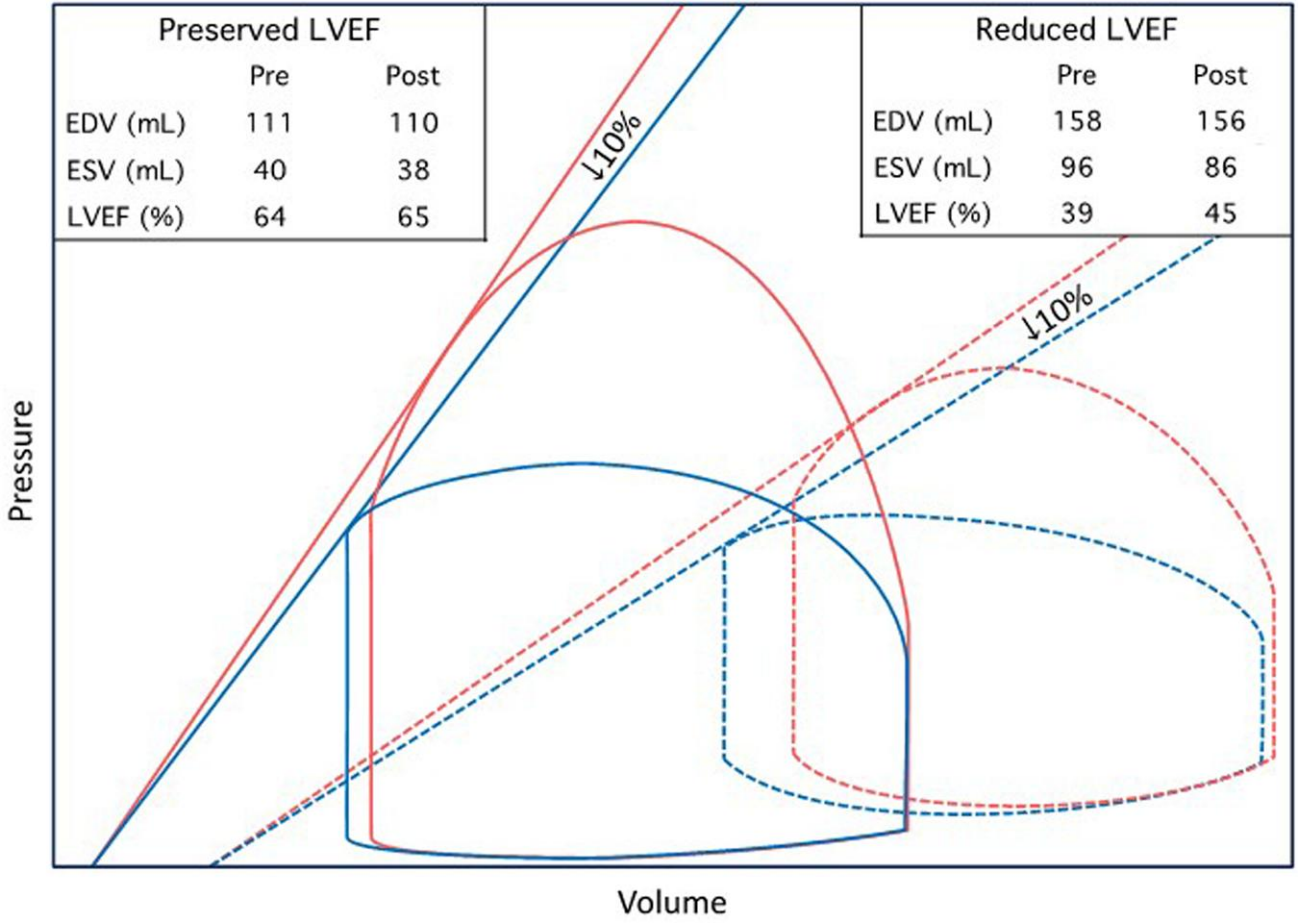
Theoretic pressure–volume diagrams depicting proposed changes in LVEF following TAVR. Two hypothetical patients with normal and abnormal LV systolic function, respectively, are illustrated. In the presence of impaired LV contractility (signified by decreased ESPVR slope), stroke volume is more sensitive to changes in afterload. Accordingly, patients with reduced LVEF experience a significant increase in LVEF following TAVR, driven by a reduction in valvuloarterial impedance, despite a 10% contractility decline. Conversely, patients with preserved baseline LVEF experience minimal or no increase despite substantial afterload reduction. Red and blue lines indicate before and after TAVR, respectively. The solid and dashed lines indicate preserved and reduced baseline LVEF, respectively.

Given that afterload is higher in AS patients with reduced LVEF (1, 27), our findings may be hypothesized to suggest positive inotropic autoregulatory mechanisms are more strongly activated in patients with reduced LVEF. Indeed, in our cohort, FSmw declined to a greater extent in patients with baseline reduced LVEF. Yet, prior studies have demonstrated an inverse relationship between baseline LVEF and the magnitude of LVEF improvement following TAVR (summarized in Table 3). Two explanations might resolve this seeming paradox. First, there was no strong correlation in our cohort between baseline wall stress and the magnitude of contractility decline; therefore, contractility augmentation in severe AS may not vary proportionately with afterload and instead occur as an “all or none” phenomenon at a certain afterload threshold. Second, the interaction between afterload and stroke volume is modified by the slope of the end-systolic pressure–volume relation (ESPVR). Patients with reduced LVEF have a higher prevalence and severity of contractile dysfunction and therefore typically experience marked improvement in LVEF with TAVR. Conversely, patients with preserved LVEF have less contractile dysfunction and steeper ESPVR slopes. Under these conditions, the impact of afterload reduction may be outweighed by an associated contractility decline, resulting in modest or no LVEF improvement. This phenomenon is illustrated in Figure 3.

**Table 3:** Summary of studies investigating changes in LVEF following TAVR.

Study	Subgroup	LVEF (% ± SD)		Time Interval	*N*	*P*-value
		Pre-TAVR	Post-TAVR			
Maes et al., 2019 (TOPAS-TAVI) (8)	LVEF <30%	22 ± 5	34 ± 12	1 year	128	<0.001
Pilgrim et al., 2011 (9)	LVEF ≤30%	25 ± 4	34 ± 10	Discharge	37	<0.001
Fraccaro et al., 2012 (10)	LVEF <35%	27.7 ± 6.0	35.4 ± 11.0	Discharge	46	<0.0001
O'Sullivan, et al., 2013 (11)	LVEF ≤40%, LFLG	29.0 ± 6	38.3 ± 13.2	30 days	20	<0.0001
O'Sullivan, et al., 2013 (11)	LVEF ≤40%, LFHG	31.8 ± 7	50.6 ± 10.0	30 days	31	<0.0001
Bauer et al., 2013 (12)	All	32 ± 9	50 ± 17	30 days	31	<0.05
Gotzmann et al., 2012 (13)	LFLG	32 ± 6	46 ± 11	6 months	15	<0.01
Van Linden et al., 2011 (14)	LVEF ≤40%	32.5 ± 7.1	41.5 ± 10.1	Discharge	39	<0.0001
			53.9 ± 13	1 year		<0.0001
Kuneman et al., 2022 (15)	LVEF <40%	33 ± 6	43 ± 10	1 year	88	<0.001
Ewe et al., 2010 (16)	LVEF <50%	37 ± 8	46 ± 11	Discharge	97	<0.001
Maes et al., 2019 (TOPAS-TAVI) (8)	LVEF 30−40%	37 ± 7	41 ± 12	1 year	165	<0.001
Elmariah et al., 2013 (PARTNER Cohort A Trial) (17)	LVEF <50%	37.1 ± 9.2	48.6 ± 11.3	1 year	108	<0.0001
Løgstrup et al., 2013 (18)	LVEF ≤50%	39 ± 9.4	52 ± 12.5	1 year	74	<0.0001
Deste et al., 2018 (19)	All	39.3 ± 8.8	44.1 ± 10.1	30 days	46	<0.05
Kuneman et al., 2022 (15)	LVEF 40–49%	45 ± 3	52 ± 8	1 year	122	<0.001
Harrington et al., 2021 (22)	LVEF <60%	45.4 ± 10	46.7 ± 9.8	1 year	NR^a^	0.15
Sato et al., 2017 (20)	All	50 ± 14	53 ± 13	10 days	209	<0.001
Popma et al., 2014 (CoreValve Extreme) (21)	All	54.5 ± 14.4	57.3 ± 11.6	1 year	330	NR
Pilgrim et al., 2011 (9)	LVEF >30%	56 ± 10	57 ± 11	Discharge	219	NR
Fraccaro et al., 2012 (10)	LVEF ≥35%	56.5 ± 8.7	55.9 ± 9.2	Discharge	315	0.24
Gotzmann et al., 2012 (13)	No LFLG	57 ± 11	56 ± 8	6 months	152	>0.05
Løgstrup et al., 2013 (18)	LVEF >50%	57.9 ± 5.3	60 ± 7.7	1 year	34	>0.05
Kuneman et al., 2022 (15)	LVEF ≥50%	58 ± 5	59 ± 7	1 year	350	<0.05
Ewe et al., 2010 (16)	LVEF ≥50%	61 ± 7	59 ± 11	Discharge	50	>0.05
Harrington et al., 2021 (22)	LVEF ≥60%	65.7 ± 5.5	53.5 ± 10.8	1 year	NR^a^	<0.01

NR, not reported; LFLG, low flow, low gradient; LFHG, low flow, high gradient.

a A total of 397 patients; distribution between reduced and preserved LVEF subgroups not available.

The variability in LVEF response to TAVR reinforces the limitations of endocardial indices in the evaluation of systolic function in the presence of altered LV geometry and loading conditions. Importantly, regardless of the impact on LVEF, withdrawal of these positively inotropic mechanisms likely restores both ventricular efficiency (i.e., the ratio of stroke work to total LV energy expenditure) and contractile reserve (i.e., the ratio of resting to maximal contractility), thereby improving exercise capacity and resistance to acute stressors, such as infection, arrhythmia, and hypovolemia (47, 48). Therefore, unlike long-term postoperative contractility impairment, which reflects poor reverse modeling and predicts poor outcomes following TAVR (49), contractility decline in the immediate term may actually be an expected and even favorable biomarker.

Several limitations of our study must be acknowledged. Our cohort was small, nonrandom, and derived from a single academic medical center, predisposing to sampling error and selection bias. While we excluded patients with clear perioperative confounders of systolic function, it is difficult to definitively rule out a preoperative contractility decline due to progression of AS-associated cardiomyopathy, particularly in patients with long time intervals between preoperative echocardiogram and TAVR. Similarly, given its procedural necessity, we were unable to exclude rapid pacing as a potential confounder of contractility. However, myocardial stunning secondary to extreme tachycardia is typically a transient and subclinical phenomenon, lasting on the order of minutes in the majority of patients (50). Accordingly, rapid pacing alone is unlikely to have caused such a profound decrease in contractility in this cohort. Lastly, Zva decreased to a lesser degree (26%) than did cESS (39%) and mean AVG (74%). Although blood pressure did not increase, this discrepancy may reflect increased arterial load, as has previously been demonstrated following TAVR (51). Therefore, afterload may be slightly underestimated by cESS following TAVR, predisposing to error during the calculation of predicted FSmw.

In summary, systolic function in severe AS seems to be supported by positive inotropic autoregulatory mechanisms. These may include increased adrenergic tone and the Anrep effect. Restoration of contractile reserve through downregulation of these mechanisms represents one of many favorable hemodynamic effects of aortic valve replacement.

## Data Availability

The original contributions presented in the study are included in the article/Supplementary Material; further inquiries can be directed to the corresponding author.
